# Parameter Optimization of Ultrasonic–Microwave Synergistic Extraction of Taxanes from *Taxus cuspidata* Needles

**DOI:** 10.3390/molecules28237746

**Published:** 2023-11-24

**Authors:** Zirui Zhao, Yajing Zhang, Wenlong Li, Yuanhu Tang, Shujie Wang

**Affiliations:** College of Biology and Agricultural Engineering, Jilin University, Changchun 130022, China

**Keywords:** ultrasonic–microwave synergistic, extraction, taxanes, parameter optimization

## Abstract

Taxanes are the best-known compounds in *Taxus cuspidata* owing to their strong anticancer effects. However, the traditional taxanes extraction method is the solid–liquid extraction method, which is limited by a large energy consumption and low yield. Therefore, it is urgent to find an efficient method for taxanes extraction. The ultrasonic microwave synergistic extraction (UME) method integrates the cavitation effect of ultrasound and the intensifying heat transfer (ionic conduction and dipole rotation of molecules) effect of microwave to accelerate the release of intracellular compounds and is used in active ingredient extractions. This study aimed to evaluate the performance of UME in extracting taxanes from *T. cuspidata* needles (dichloromethane-ethanol as extractant). A single-factor experiment, Plackett–Burman design, and the response surface method showed that the optimal UME parameters for taxanes extraction were an ultrasonic power of 300 W, a microwave power of 215 W, and 130 sieve meshes. Under these conditions, the taxanes yield was 570.32 μg/g, which increased by 13.41% and 41.63% compared with the ultrasound (US) and microwave (MW) treatments, respectively. The reasons for the differences in the taxanes yield were revealed by comparing the physicochemical properties of *T. cuspidata* residues after the UME, US, and MW treatments. The cell structures were significantly damaged after the UME treatment, and numerous tiny holes were observed on the surface. The absorption peaks of cellulose, hemicellulose, and lignin increased significantly in intensity, and the lowest peak temperature (307.40 °C), with a melting enthalpy of −5.19 J/g, was found after the UME treatment compared with the US and MW treatments. These results demonstrate that UME is an effective method (570.32 μg/g) to extract taxanes from *T. cuspidata* needles by destroying cellular structures.

## 1. Introduction

*Taxus cuspidata*, belonging to the yew family, is an endangered and slow-growing evergreen shrub or tree with ornamental and medicinal values. Taxanes are among the active ingredients in the needles, barks, and branches of *T. cuspidata* [1]. Of the taxanes, paclitaxel as a diterpenoid is the most important compound. This is because paclitaxel can inhibit cell mitosis through the formation of highly stable microtubules [2], and has been widely used to treat breast, ovarian, lung, small intestine, and other cancers since being isolated from the bark of *T. brevifolia* in 1960 [3,4]. The increasing clinical demand for paclitaxel has promoted the development of different methods, such as chemical synthesis, chemical semi-synthesis, cell culture, endophytic fungal synthesis, and metabolic engineering. However, all of the above methods have drawbacks, such as low yields, high production costs, complex processes, and the unstable expression of cell lines [2]. Currently, industrial paclitaxel production relies on the direct extraction or semi-synthesis method [5]. The reactive materials of the semi-synthesis method can also be obtained by extracting paclitaxel precursors (e.g., 10-deacetylbaccatin III (10-DAB III), baccatin III, 10-desacetylpaclitaxel (10-DAT), cephalomannine) from the needles, bark, and branches of *Taxus*. For this purpose, the traditional method is solid–liquid extraction, which has low productivity, a long processing time, and large organic solvent consumption [6]. With the ultrasonic method and microwave method, the optimal taxanes treatment times are 1.11 h [7] and 10 min [8], respectively. The dichloromethane–ethanol solvent is the most utilized extractant owing to its high solubility for taxanes [7]. Therefore, it is urgent to find a new method for taxanes extraction.

Ultrasound can generate mechanical and cavitative effects to rapidly release intracellular active ingredients. Under ultrasonic conditions, the mass transfer property is enhanced because the solvent penetrates more easily into the cells with porous surfaces. Moreover, the micro-jet, high pressure, and high temperature caused by cavitation bubbles collapse when biological cells rupture the plant matrix during the compression cycle [9]. Microwaves are electromagnetic waves that burst cellular structures based on the heat irradiation produced by their interaction with polar molecules. Under microwave conditions, charge carriers electrophoretically migrate, and dipolar molecules are rotated to maintain a similar electric field orientation, thus converting kinetic energy into thermal energy [10]. Ultrasonic microwave synergistic extraction (UME) is an efficient, cost-effective, novel extraction method because it integrates the cavitation effect of ultrasound and the intensifying heat transfer (ionic conduction and dipole rotation of molecules) effect of microwaves [11,12]. Therefore, the inhomogeneous mass and heat transfer distribution problems faced by microwaves are compensated by the cavitation effect of ultrasound. In addition, cell fragmentation and active substance release are promoted under a seamless interaction between the ultrasound-induced cavitation bubbles and the microwave-granted high temperature [9,10,13]. For the active ingredients with poor polarity and thermal stability, UME avoids decomposition and causing structural damage to these compounds due to a shorter treatment time [14]. In addition, this phenomenon is associated with the extract properties, dielectric constant and microwave. The work principle of microwaves is the ionic conduction and dipole rotation of molecules. Under microwave conditions, the chemical bonds of polar substances vibrate and tear, and the friction and collision occurring between polar particles lead to the degradation of polar substances. However, non-polar or poorly polarized compounds do not absorb microwave energy, and thermal irradiation and solution agitation promote their diffusion in the medium. Based on these advantages, many scholars have applied UME to extract different substances. For instance, Xu et al. extracted polysaccharides from *Morchella conica* using UME and found that the extraction rate was significantly increased in comparison with ultrasound or microwave treatment [15]. Estrada-Gil et al. also found that UME exhibited the highest rate in extracting polyphenols from rambutan byproduct peels compared with ultrasound or microwave treatment [10]. Kwansang et al. reported that UME can extract bioactive substances from rambutan peels with high efficiency, and optimized the extraction parameters [16]. Nevertheless, taxanes extraction from the needles of *T. cuspidata* using UME has not been reported yet.

In this study, we aimed to extract five representative taxanes (baccatine III, 10-DAB III, 10-DAT, cephalomannine, and paclitaxel) from the needles of *T. cuspidata* using UME. The process parameters were optimized through a single-factor test, the Plackett–Burman design (PBD), and the response surface method (RSM). Based on the optimized UME process conditions, the effects of ultrasonic extraction without microwave treatment (US), microwave extraction without ultrasound treatment (MW), and UME on the taxanes yield and the physicochemical properties of residues were compared.

## 2. Results and Discussion

### 2.1. Single-Factor Experiments

According to the pre-experiment results, the control values of other factors, including the ultrasonic power, microwave power, treatment time, extraction temperature, solid–liquid ratio, extraction time, and sieve mesh number, were fixed at 300 W, 200 W, 120 s, 50 °C, 1:60, 2, and 120 mesh, respectively, when a single factor was changed. The dichloromethane–ethanol solution (volume ratio of 1:1) was employed as the extraction solution.

#### 2.1.1. Ultrasonic Power

**Figure 1:**
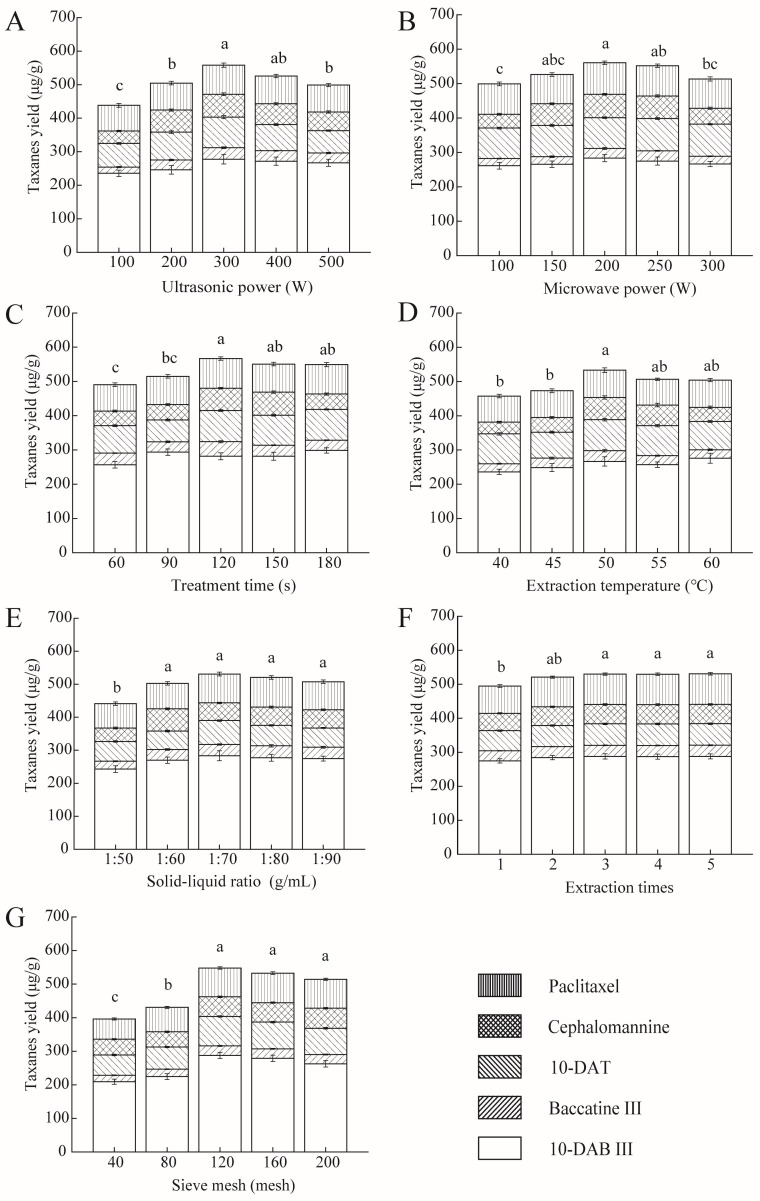
Effect of the ultrasonic power (**A**), microwave power (**B**), treatment time (**C**), extraction temperature (**D**), solid–liquid ratio (**E**), extraction times (**F**), and sieve mesh number (**G**) on the taxanes yield. All data are presented as mean ± standard deviation. Data were collected from three independent experiments and analyzed using one-way analysis of variance. Different letters (a, b, c) in the same figure indicate significant differences (*p* < 0.05).

We firstly investigated whether UME was feasible for taxanes extraction. Power is an important factor affecting the effectiveness of UME treatment, and includes ultrasonic power and microwave power. Therefore, ultrasonic power was first chosen to explore the taxanes yield of the UME treatment. The probe diameter, probe surface area, ultrasound frequency, maximum processing volume, and maximum ultrasonic power of the UME equipment employed were 6 mm, 0.282 cm^2^, 25 kHz, 500 mL and 900 W, respectively. The effects of ultrasonic power on the taxanes yield are shown in Figure 1A. With the increment in ultrasonic power from 100 to 300 W, the taxanes yield increased from 438.38 to 558.12 μg/g. This was because the organic solvent penetration and taxanes dissolution were accelerated by the cavitation effect and mechanical vibration of ultrasound. Specifically, the hydroxyl and hydrogen bonds formed between the extraction solvent and the plant cellulose were continuously disrupted by the ultrasonic treatment, which enlarged the concentration difference between the intracellular and extracellular media. In addition, the “cavitation” produced by ultrasound in liquids destroyed the plant cells and cell membrane structures [17]. However, the taxanes yield decreased under the highest ultrasound power (500 W), which may be due to the structure damage or degradation of taxanes [18]. The higher prejudicial yield of 10-DAT beyond the ultrasonic power of 300 W compared with other taxanes may be attributed to the largest amount of hydroxyl (3) in the structure of 10-DAT compared with other taxanes, which increased the polarity of the compounds. These speculations will be further explored in our next study.

#### 2.1.2. Microwave Power

The effect of microwave power on the taxanes yield is shown in Figure 1B. With the increase in microwave power from 100 to 200 W, the taxanes yield subsequently rose from 499.17 to 560.66 μg/g. The reason for this trend was that the microwave radiation induced a high-energy input in the electromagnetic field by vibrating and tearing the chemical bonds of the polar substances, thus facilitating the penetration of solvents into the powder [19]. In addition, the overlap of the heat and mass transfer direction with full heat coverage accelerated the transfer efficiency [17]. However, the taxanes yield declined beyond 200 W, indicating that a higher microwave power does not guarantee higher extraction yields [12]. The reason for this phenomenon may be due to the thermal degradation of taxanes. Plant materials will be carbonized at a high irradiation power because of internal overheating, isomerization, or thermal instability [20]. Damage to the structure and function of oligosaccharides at high temperatures was also reported [21]. Under the electromagnetic field generated by microwave radiation, ionic conduction and dipole rotation will occur between dichloromethane and ethanol, because both substances are polar, and their permittivities are 9.08 and 24.3 F/m, respectively. The dipole moment values are 1.60 and 5.61 D, respectively. All these moving molecules generate heat rapidly, which degrades the taxanes and decreases the extraction yields, owing to the thermal sensitivity of taxanes [22,23]. Exploratory research will be performed in the next study.

#### 2.1.3. Treatment Time

The treatment time is another important factor affecting the extraction effects [11]. The taxanes yield increased with the prolonged treatment time and reached 566.82 μg/g at 120 s (Figure 1C). However, the extraction yield did not significantly change with a further increase in the treatment time (*p* > 0.05). Similar results were reported in flavonoid extraction and total polyphenol extraction [20,24]. The underlying reason may be that taxanes were completely extracted at 120 s. Compared to the treatment time required in taxanes extraction with ultrasound (47.63 min) [25] or microwave (10 min) [8], the treatment time required in this study was significantly shortened (120 s). Furthermore, the taxanes yield was 351.93 ± 31.83 μg/g after 2 h of treatment with traditional solid–liquid extraction [26]. The shorter consumption time with a higher extraction efficiency can be explained firstly by the fact that the mass and heat transfer direction of UME overlapped, but certainly also by compensation for the inhomogeneity of microwave heat transfer via the action of ultrasound. Therefore, the energy utilization and extraction efficiency were improved, and the treatment time was shortened.

#### 2.1.4. Extraction Temperature

For thermosensitive compounds, the extraction temperature is a critical factor affecting the extraction effect. With a rise in the extraction temperature, the solvent diffusion and the dissolution of the target compounds were accelerated. When the extraction temperature rose from 40 to 50 °C, the taxanes yield increased from 457.43 to 533.00 μg/g (Figure 1D). A similar phenomenon was observed by Wianowska et al. [27] when extracting paclitaxel from *T. brevifolia* using a pressurized liquid-phase technique. However, the taxanes yield decreased when the extraction temperature exceeded 50 °C, which may be due to the degradation of taxanes [28]. Significant taxanes degradation was observed at temperatures above 50 °C [8].

#### 2.1.5. Solid–Liquid Ratio

In the solid–liquid extraction, the yield of active ingredients rose with the increasing solvent volume until reaching the equilibrium point. To maximize the extraction yield with the least solvent consumption, it is necessary to study the effect of the solid–liquid ratio on taxanes extraction [29]. The taxanes yield rose as the solvent volume increased from 50 to 70 mL/g (Figure 1E). The highest value was obtained (530.88 μg/g) at the solid–liquid ratio of 1:70. This may be because a low volume of solvents cannot completely extract taxanes from cells. As the solvent volume increased, the concentration difference between the intracellular and extracellular media increased, which promoted the diffusion and solubilization of the target products. However, as the solvent volume further increased, the taxanes yield no longer significantly changed (*p* > 0.05). Similar findings were reported by Fan et al. [12]. This is because the excessive solvent weakened the destructive effect of ultrasound and microwave on the samples. In addition, a large solvent consumption can cause waste and increase costs.

#### 2.1.6. Number of Extractions

Since increasing the extraction number improves the partition of active ingredients in the extraction solvent, the number of extractions is usually increased in traditional solvent extraction methods [30]. To explore whether UME requires multiple extractions, the effect of the extraction number on the taxanes yield was investigated. *T. cuspidata* needle powders filtered through a 120-mesh sieve (2 g) were placed into a three-neck flask, and 120 mL of dichloromethane ethanol was added as the solvent. The ultrasonic power, microwave power, extraction time, temperature, and the sieve mesh number were set at 300 W, 200 W, 120 s, 50 °C, and 120 meshes, respectively. After the first extraction, the solution was filtered, and the extraction process was repeated four times consecutively. The filtrates were combined to measure the taxanes yield. The results are shown in Figure 1F. With the increase in extraction times, the taxanes yield rose significantly from one to three and then remained stable. The taxanes yields after one, two, and three extractions were 494.83, 520.91, and 529.95 μg/g, respectively, indicating that there were no more taxanes in the residues after two extractions via the UME. Similar results were found by Luo et al. [8], who detected no taxanes after the 4th and 5th extractions using MW.

#### 2.1.7. Sieve Mesh Number

The effect of the sieve mesh number on the taxanes yield is shown in Figure 1G. With the increment in the sieve mesh number, the taxanes yield increased rapidly at first and then remained stable. The maximum taxanes yield was obtained at 120 meshes (547.92 μg/g), indicating that increasing the sieve mesh number can improve the active ingredient yield. Similarly, Kwansang et al. demonstrated that a powder diameter of 22.6 μm outperformed that of 75.3 μm when extracting total phenols, caffeic acid, and ferulic acid from mangosteen pericarp with UME [16]. The larger sieve mesh number led to a smaller powder particle size. The powder surface area was enlarged at the same time, which promoted the contact of the powder with the solvent, and increased the active ingredient extraction [18].

### 2.2. Analysis of PBD Results

PBD can filter out a few important variables from multiple factors [31]. PBD was employed to screen the key factors of taxanes extraction with UME. Each factor was set at two levels (lowest and highest) according to the single-factor experiments [32]. The PBD results are shown in Table 1.

**Figure 2:**
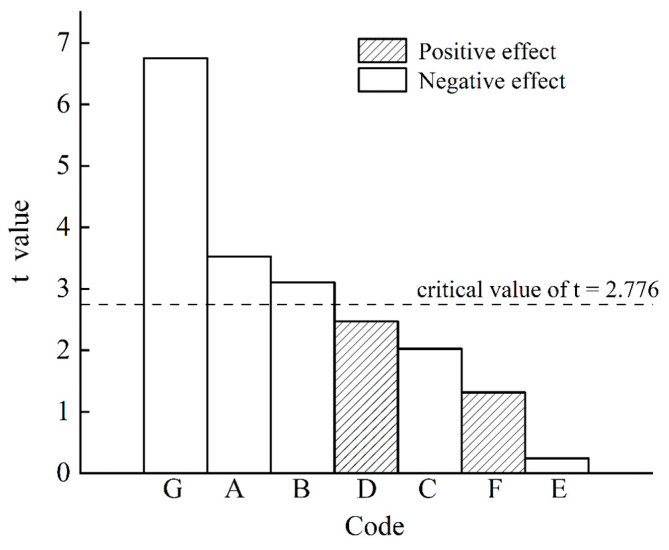
Pareto plot of the normalized effects of each variable. A: ultrasonic power; B: microwave power; C: treatment time; D: treatment temperature; E: solid–liquid ratio; F: extraction times; G: sieve mesh number.

The results of the analysis of variance (ANOVA) are shown in Table 2. According to the *p* values of the seven tested parameters, the ultrasonic power (A), microwave power (B), and sieve mesh number (G) all significantly affected the taxanes yield (*p* < 0.05), while other parameters did not contribute significantly to the taxanes yield (*p* > 0.05). The Pareto plots of the variables are shown in Figure 2. All the significant parameters, including the ultrasonic power (A), microwave power (B), and sieve mesh number (G), positively affected the taxanes yield (Figure 2). Hence, these three factors were selected for further optimization experiments.

The taxanes yield model developed from the PBD is Y = 487.47 + 15.91A + 14.00B + 9.13C − 11.15D + 1.08E − 5.93F + 30.45G. The *p* value of the regression equation is 0.0167. The correlation coefficient (R^2^) of the model is 0.9521. The adjusted R^2^ (adj R^2^) is 0.8683, and the signal-to-noise ratio is 3.21, indicating a good fit for this model.

### 2.3. Analysis of Central Composite Design (CCD) Results

According to the PBD results, a CCD was implemented, with the taxanes yield as the response (Y, μg/g) and the ultrasonic power (X_1_), microwave power (X_2_), and sieve mesh number (X_3_) as variables. The results are shown in Table 3. Other parameters, including the treatment time, treatment temperature, solid–liquid ratio, and extraction number, were fixed at 120 s, 50 °C, 1:60, and 2 times, respectively.

To investigate the influence of the parameters and their interactions on the taxanes extraction, the data in Table 4 were fitted via multiple regression using Design Expert 10. The quadratic multiple regression equation was obtained as follows:

Y = 569.98 + 11.07X_1_ + 10.56X_2_ + 19.41X_3_ − 5.01X_1_X_2_ − 17.39X_1_X_3_ + 12.35X_2_X_3_ − 28.98X_1_^2^ − 22.86X_2_^2^ − 32.90X_3_^2^ (1)

where X_1_, X_2_, and X_3_ denote the coded variables of ultrasonic power, microwave power, and sieve mesh number, respectively; and Y is the total concentration of the five main taxanes, μg/L.

The ANOVA of the RSM model is shown in Table 4. The regression model is highly significant (*p* < 0.0001), but the out-of-fit phase is not significant, with R^2^ of 0.9741, indicating that the model fits well and can reasonably predict the taxanes yield within the ranges of the variables [33]. The most important factor affecting the taxanes yield is the sieve mesh number, which is consistent with the results of the PBD.

**Figure 3:**
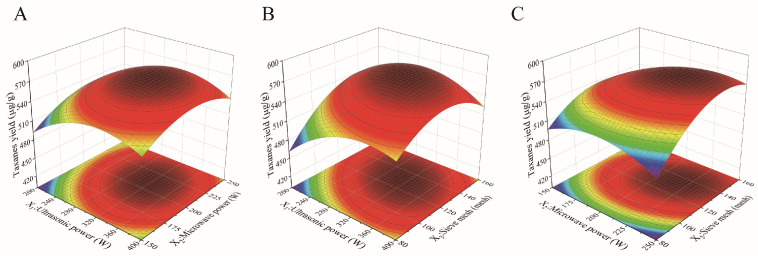
Response surface for the effects of ultrasonic power, microwave power, and sieve mesh number on the extraction yield of taxanes. (**A**) ultrasonic power (X_1_) and microwave power (X_2_); (**B**) ultrasonic power (X_1_) and sieve mesh number (X_3_); (**C**) microwave power (X_2_) and sieve mesh number (X_3_).

To examine the effects of the tested factors on the taxanes yield, response surface plots were used to characterize the three-dimensional relationships between the respective variables and the response value (Figure 3). A steeper shape in the response surface plot means that the interaction of variables is more pronounced. The strongest and weakest interactions were found between ultrasonic power and the sieve mesh number (Figure 3B), and between ultrasonic power and microwave power (Figure 3A). With the increase in ultrasonic power and sieve mesh number, the taxanes yield rose first and then decreased (Figure 3B), which is consistent with the results of the single-factor experiments.

The adjusted optimal parameters of taxanes extraction with UME were obtained by calculating the multiple regression equation and combining the operability of the actual experiment: ultrasonic power of 300 W, microwave power of 215 W, treatment time of 120 s, extraction temperature of 50 °C, solid–liquid ratio of 1:60, two extractions, and 130 sieve meshes. Under these conditions, the actual yield of taxanes was 570.32 ± 29.53 μg/g (Table 5). The *t*-test revealed no significant difference between the actual and predicted values (574.81 μg/g), which indicates the reliability of the RSM model.

### 2.4. Comparison of Different Extraction Methods

#### 2.4.1. Taxanes Yield

The taxanes yield of *T. cuspidata* needles after different treatment methods was calculated (Table 5). The highest taxanes yield (570.32 μg/g) was obtained via UME, and increased by 41.63% and 13.41% compared with the US and MW methods respectively, indicating that UME has a higher extraction efficiency.

#### 2.4.2. Surface Morphology (SEM)

**Figure 4:**
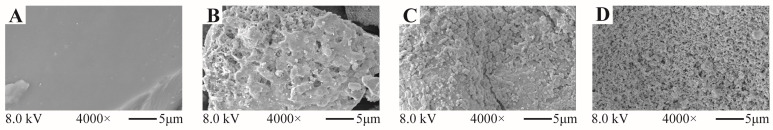
SEM images of *T. cuspidata* residues with different treatments. (**A**) untreated sample, (**B**) ultrasonic extraction, (**C**) microwave extraction, (**D**) ultrasonic–microwave synergistic extraction.

The extraction yields are closely related to the alteration of cell microstructures. The surface structural features of the samples are shown in Figure 4. Clearly, the surface of the untreated sample is smooth and intact (Figure 4A). After US treatment, the cell surface becomes uneven with holes (Figure 4B). Specifically, the cellular structure in the upper-left corner of Figure 4B was completely destroyed, with an uneven surface and many small holes. The cellular structure in the lower-right corner was less damaged than that in the upper-left corner, but also showed larger holes. This was because the cavitation effect of ultrasound destroyed the plant cells. After MW treatment, many protrusions of spherical structures appeared on the surface of the samples (Figure 4C). This may be because the sudden increase in temperature and internal pressure led to the rapid exudation of intracellular materials and the rupture of the cell wall structures [20]. The UME treatment considerably affected the plant cell structures (Figure 4D). The samples had a spongy structure, with many tiny pores and weak connections. These phenomena may be caused by the synergistic effect of ultrasound and microwaves. As the microwaves increased the internal pressure of cells, the mechanical and cavitation effects of ultrasound accelerated the rupture of cell walls and cell membranes, which quickened the heat and mass transfer. As a result, the morphology of the UME-treated samples changed. Xu et al. also observed that the numbers of pores and cracks on the mushroom cell surface increased significantly after UME treatment [15].

#### 2.4.3. Fourier-Transform Infrared Spectroscopy (FTIR)

**Figure 5:**
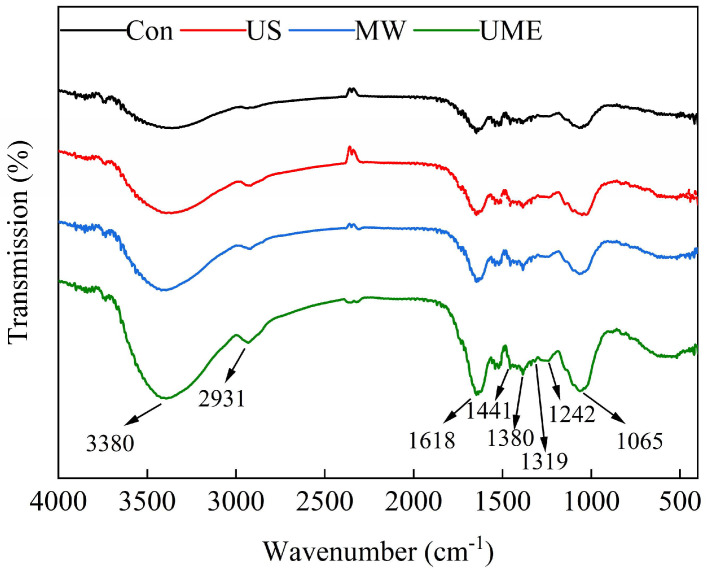
Infrared spectra of *T. cuspidata* residues with different treatments. (Con) control group, (US) ultrasonic treatment, (MW) microwave treatment, and (UME) ultrasonic–microwave synergistic treatment.

FTIR is a powerful technique used to characterize organic functional groups. The cell wall of wood is mainly composed of three components, namely cellulose, hemicellulose, and lignin, and its infrared spectral characteristics directly reflect the changes in these three components. The FTIR spectra of the residues after different treatments are shown in Figure 5. The absorption peak near 3380–3400 cm^−1^ reflects the O-H stretching vibration of cellulose [34]. The peak at 2931 cm^−1^ stands for the C-H stretching vibrations of methyl, methylene, and methyne groups, which are the common absorption peaks of cellulose and lignin [35]. The peak at 1618 cm^−1^ is ascribed to the antisymmetric stretching vibration of carboxylic acid (COO-) or the stretching vibration of the aromatic ring (C=C) in lignin. The peak at 1441 cm^−1^ is caused by the -CH_2_ and -CH_3_ stretching oscillations on the carboxyl and ester groups in cellulose and lignin. The peaks near 1380 and 1242 cm^−1^ are induced by terpene skeleton vibrations. The peaks at 1380 and 1319 cm^−1^ also reflect the C-H bending and stretching vibrations of the glucose units in cellulose and hemicellulose. The broad and strong peak near 1065 cm^−1^ is ascribed to the C-OH bending vibration of glycosides or the C-O-C stretching vibration of cellulose and hemicellulose.

Compared with the Con group, the characteristic absorption peaks of the samples treated with US, MW, and UME increased in intensity, indicating that the functional groups (C=O, C-O-C, C-OH, and C-H) of cellulose, hemicellulose, and lignin were exposed. Since cellulose, hemicellulose, and lignin are the main constituents of cell walls, the revelation of these groups reflects cell wall fragmentation, which eliminates selective permeation and promotes intracellular substance leaching. These phenomena are consistent with another study reporting the degraded cellulose, hydrolyzed hemicellulose, and lignin of *T. cuspidata* needles after high-pressure extraction [36]. Li et al. also observed the higher absorption intensity of haskap powder and a higher anthocyanin yield after cold plasma treatment, which were attributed to the breakdown of cell walls (pectin, hemicellulose, and cellulose) [37].

#### 2.4.4. Thermal Properties

DSC was employed to reflect the temperature and enthalpy transitions in the samples after different treatments. The onset, end and peak temperatures, and melting enthalpy (∆H) are presented in Table 6. All samples showed a heat absorption peak near 310 °C, indicating the occurrence of crystal melting at that location, such as the melting absorption peak of polysaccharide crystals (cellulose, hemicellulose, and lignin) or carbohydrate degradation [38]. The peak temperatures of Con, US, MW, and UME were 314.54, 310.65, 310.01, and 307.40 °C, respectively. Differences in T_peak_ indicate no homogeneity in the residue structures [38]. In addition, the absolute values of ∆H in Con, US, MW, and UME were 7.92, 5.86, 5.69, and 5.19 J/g, respectively. The highest values of T_peak_ and ∆H were observed in the Con samples and the lowest were observed in the UME treatment. This is probably related to the contents of cellulose, hemicellulose, and lignin. As the destruction degree of cellulose, hemicellulose, and lignin increases, the specific surface areas of the samples are enlarged, and the energy and temperature required for degradation are reduced [38]. Similarly, Guo et al. showed that the endothermic temperature of cellulose decreased after treatment with HCl or H_2_SO_4_ [39]. Moczkowska et al. also observed the lowest T_peak_ of soluble dietary fiber after enzymatic-assisted ultrasound treatment at 55 °C [40].

## 3. Materials and Methods

### 3.1. Materials

The needles of *T. cuspidata* were harvested from Changbai Mountain, Jilin, China. These samples were cleaned, dried at 40 °C, crushed, sieved, and stored in a dry environment. Standard Baccatine III, 10-DAB III, 10-DAT, Cephalomannine, and Paclitaxel were purchased from Yuanye Biotechnology Co., Shanghai, China. The other reagents, such as dichloromethane and ethanol, were bought from Damao Chemical Reagent Factory, Tianjin, China, all of which were of analytical grade.

### 3.2. Taxanes Extraction

According to the pre-experimental results, 2.0 g of dried needle powder was added to a three-necked round-bottomed flask with 120 mL of a dichloromethane–ethanol solution (volume ratio of 1:1). Then, the flask was put into a UME apparatus (XO-SM50, Xianou Instrument Co., Ltd., Nanjing, China) equipped with a microwave apparatus (maximal microwave power of 700 W at a frequency of 2450 MHz) and an ultrasonic transducer with a fixed frequency of 25 kHz. The probe diameter, probe surface area, maximum processing volume, and maximum ultrasonic power of the UME apparatus were 6 mm, 0.282 cm^2^, 500 mL and 900 W, respectively. This UME apparatus was equipped with a circulating chiller system, with its temperature controlled at −40 to 500 °C. The treatment time was set at 120 s, and the extraction temperature was 50 °C. After extraction, the supernatant and sediment were collected via centrifugation and filtration, respectively. The centrifugation temperature, duration and speed were set at 4 °C, 5000 rpm, and 10 min, respectively. Filtration was performed using a 0.22 μm polyvinylidene fluoride filter membrane. The supernatant was evaporated in a rotary evaporator and then re-dissolved with 3 mL of methanol. After that, the solution was passed through a 0.22 μm nylon filter membrane for measurement.

### 3.3. Measurement of Taxanes

The taxanes were detected via high-performance liquid chromatography (HPLC) according to Zhao et al. [30] and Fan et al. [6]. 10-DAB III, baccatin III, 10-DAT, cephalomannine, and paclitaxel were weighed accurately and made into 1 mg/mL standard solutions separately. Then, mixed standard solutions of 1, 2, 5, 10, and 100 mg/L were obtained, and standard curves were drawn (Figure S1). The regression equation and linear range of the five main taxanes are shown in Table S1. The HPLC was operated with a Waters C18 column (250 × 4.6 mm, 5 μm) at a flow rate of 1.0 mL/min. The injection volume was 10 μL, the column temperature was 30 °C, and the detection wavelength was 227 nm. Acetonitrile and ultrapure water were used as mobile phases A and B, respectively. The gradient elution program was as follows: mobile phase A from 40% to 50% at 0–10 min, from 50% to 53% at 10–13 min, from 53% to 73% at 13–25 min, and from 73% to 40% at 29–40 min. The taxanes yield (Y) in the needles of *T. cuspidata* was calculated according to the standard curves.

Y = C × V/M,(2)

where C is the total concentration of the five main taxanes, μg/L; V is the volume of the extraction solution, mL; and M is the mass of *T. cuspidata*, g.

### 3.4. Single-Factor Experiments

According to the pre-experimental results, the ultrasonic power (100, 200, 300, 400, 500 W), microwave power (100, 150, 200, 250, 300 W), treatment time (60, 90, 120, 150, 180 s), extraction temperature (40, 45, 50, 55, 60 °C), solid–liquid ratio (1:50, 1:60, 1:70, 1:80, 1:90), and extraction cycle number (1, 2, 3, 4, 5) were selected, and all were set at five levels to obtain the appropriate level of each factor. The control values of ultrasonic power, microwave power, treatment time, extraction temperature, solid–liquid ratio, extraction time, and sieve mesh number were fixed at 300 W, 200 W, 120 s, 50 °C, 1:60, 2, and 120 meshes, respectively.

### 3.5. PBD

Based on the results of the single-factor experiments, the PBD was employed to find the significant factors affecting the taxanes yield among the seven factors (*n* = 12). The highest and lowest levels of each factor were selected according to the results of the single-factor experiments.

### 3.6. CCD

According to the results of the PBD, a response surface model (RSM) was designed using the principle of CCD. In this design, the ultrasonic power (X_1_), microwave power (X_2_), and sieve mesh number (X_3_) were selected as independent variables, and the taxanes yield (Y) was set as the response variable (Table 4). The experiment design, modeling, and data analysis were accomplished using Design-Expert 10 [41].

### 3.7. Scanning Electron Microscopy (SEM)

The microstructures of the residues after UME treatment, US treatment and MW treatment and those of the untreated samples (Control) were observed by an SEM meter (Sigma 300, Carl Zeiss, Oberkochen, Germany). The air-dried samples were coated on black conductive adhesive and fixed on a specimen holder. The samples were made conductive via gold sputtering before observation. The samples were observed at a high pressure of 8.0 kV and photographed at 4000×.

### 3.8. FTIR of Residues

Each sample (10 mg) mixed with 100 mg of potassium bromide was compressed into salt disks (10 mm in diameter), which were observed using an FTIR meter (VERTEX 70, Bruker Corporation, Karlsruhe, Germany). The scanning number and resolution were set at 32 and 2 cm^−1^, respectively.

### 3.9. Thermal Property Analysis of Residues

Residues of each sample (8 mg) after different treatments were placed in a crucible for the analysis of thermal properties using a TA Q20 differential scanning calorimeter (DSC, TA Instruments, Delaware, America). The heating speed and nitrogen flow rate were set at 10 °C /min and 50 mL/min, respectively.

### 3.10. Statistical Analysis

Figures were drawn using Origin 2019. The PBD and RSM were analyzed using Minitab 19.0 and Design-Expert 10, respectively. Significant differences between samples (*p* < 0.05) were analyzed using Duncan’s multiple range test. ANOVA was conducted using SPSS 25.0. All experiments were performed three times, and the results were expressed as mean ± standard deviation.

## 4. Conclusions

UME is an effective way to extract taxanes from *T. cuspidata* needles. The best parameters obtained via the single-factor test, PBD, and CCD are as follows: ultrasonic power of 300 W, microwave power of 215 W, treatment time of 120 s, extraction temperature of 50 °C, solid–liquid ratio of 1:60, two extractions, and a sieve mesh number of 130. Under these conditions, the highest taxanes yield (570.32 μg/g) was obtained, which increased by 13.41% and 41.63% compared with the US and MW methods, respectively. The SEM, FTIR, and thermal properties of the residues from *T. cuspidata* needles after UME showed that cell fragmentation increased, and that the characteristic groups of cellulose, hemicellulose, and lignin were exposed. The lowest thermal stability was observed compared with the US and MW treatments. In conclusion, UME is a promising method, with the potential to extract active ingredients from other plant materials; it also provides an idea for fully exploring the effects of process parameters on extraction yields.

## Figures and Tables

**Table 1:** Results of the Plackett–Burman assay.

	A (W)	B (W)	C (s)	D (°C)	E (g/mL)	F	G (Mesh)	Extraction Yields (μg/g)
1	1 (400)	−1 (150)	1 (150)	1 (55)	−1 (1:50)	1 (3)	1 (160)	510.37
2	1	−1	1	1	1 (1:70)	−1 (1)	−1 (80)	453.56
3	−1 (200)	1 (250)	−1 (90)	1	1	−1	1	488.16
4	1	−1	−1	−1 (45)	1	−1	1	541.87
5	1	1	−1	1	1	1	−1	473.73
6	−1	1	1	1	−1	−1	−1	470.95
7	−1	−1	−1	1	−1	1	1	461.16
8	−1	−1	1	−1	1	1	−1	438.77
9	−1	1	1	−1	1	1	1	535.19
10	1	1	1	−1	−1	−1	1	570.77
11	1	1	−1	−1	−1	1	−1	470.00
12	−1	−1	−1	−1	−1	−1	−1	435.08

A: ultrasonic power; B: microwave power; C: treatment time; D: treatment temperature; E: solid–liquid ratio; F: extraction times; G: sieve mesh number.

**Table 2:** ANOVA for PBD experiments.

Origin	Square Sum of Dispersion	df	Mean Square	F Value	*p* Value
Model	19,446.64	7	2778.09	11.36	0.0167
A	3039.35	1	3039.35	12.43	0.0243
B	2351.08	1	2351.08	9.62	0.0362
C	1001.29	1	1001.29	4.10	0.1130
D	1490.55	1	1490.55	6.10	0.0690
E	13.99	1	13.99	0.057	0.8227
F	422.06	1	422.06	1.73	0.2592
G	11,128.32	1	11,128.32	45.52	0.0025
Residual	977.90	4	244.47		
Cor total	20,424.54	11			

A: ultrasonic power; B: microwave power; C: treatment time; D: treatment temperature; E: solid–liquid ratio; F: extraction times; G: sieve mesh number.

**Table 3:** Experimental design and results of CCD.

No.	Ultrasonic Power (X_1_)	Microwave Power (X_2_)	Sieve Mesh Number (X_3_)	Extraction Yields (Y)
1	1.682 (468)	0 (200)	0 (120)	502.72
2	0 (300)	−1.682 (116)	0	496.97
3	0	0	0	573.99
4	0	0	0	560.99
5	0	0	−1.682 (50)	440.85
6	1 (400)	1 (250)	1 (160)	518.84
7	−1 (200)	−1 (150)	−1 (80)	427.04
8	0	0	0	559.86
9	0	0	0	574.76
10	−1.682 (132)	0	0	479.65
11	0	0	0	563.23
12	0	0	1.682 (190)	519.32
13	0	1.682 (284)	0	520.03
14	−1	−1	1	474.47
15	0	0	0	585.97
16	−1	1	−1	442.80
17	1	−1	−1	504.03
18	1	−1	1	473.75
19	1	1	−1	491.59
20	−1	1	1	531.47

**Table 4:** ANOVA for response surface polynomial model.

Sources	Sum of Squares	df	Mean Square	F Value	*p* Values
Model	41,781.48	9	4642.39	41.83	<0.0001
X_1_	1674.67	1	1674.67	15.09	0.0030
X_2_	1522.72	1	1522.72	13.72	0.0041
X_3_	5143.59	1	5143.59	46.34	<0.0001
X_1_X_2_	201.11	1	201.11	1.81	0.2080
X_1_X_3_	2419.82	1	2419.82	21.80	0.0009
X_2_X_3_	1219.46	1	1219.46	10.99	0.0078
X_1_^2^	12,101.65	1	12,101.65	109.04	<0.0001
X_2_^2^	7529.60	1	7529.60	67.84	<0.0001
X_3_^2^	15,602.74	1	15,602.74	140.58	<0.0001
Residual	1109.85	10	110.99		
Lack of fit	586.63	5	117.33	1.12	0.4516
pure error	523.22	5	104.64		
Cor total	42,891.33	19			

X_1_: ultrasonic power; X_2_: microwave power; X_3_: sieve mesh number; *p* < 0.05, the difference is significant; *p* < 0.01, the difference is extremely significant.

**Table 5:** The content of five compounds after being extracted with different methods.

Methods	Yield (μg/g)					
	10-DAB III	Baccatine III	10-DAT	Cephalomannine	Paclitaxel	Taxanes
US	271.38 ± 9.37 ^b^	31.69 ± 1.75 ^a^	78.45 ± 6.58 ^b^	44.10 ± 1.96 ^b^	77.25 ± 6.34 ^a^	502.87 ± 26.00 ^b^
MW	230.31 ± 7.28 ^c^	25.54 ± 1.83 ^b^	52.05 ± 2.09 ^c^	37.35 ± 2.21 ^c^	57.43 ± 5.35 ^b^	402.68 ± 18.76 ^c^
UME	289.78 ± 9.55 ^a^	31.70 ± 1.79 ^a^	98.60 ± 2.11 ^a^	59.45 ± 2.46 ^a^	90.79 ± 13.62 ^a^	570.32 ± 29.53 ^a^

US: ultrasonic extraction, MW: microwave extraction, UME: ultrasonic–microwave synergistic extraction, Taxanes: taxanes equivalents of *T. cuspidate* (μg/g), 10-DAB III: 10-deacetylbaccatinIII, 10-DAT: 10-deacetyltaxol. Different letters in the same column indicate statistically significant differences according to Duncan’s multiple range test (*p* < 0.05).

**Table 6:** DSC results of *T. cuspidata* needle residues after different treatments.

Treatment	T_on_ (°C)	T_end_ (°C)	T_peak_ (°C)	∆H (J/g)
Con	298.40 ± 0.13	326.12 ± 0.42	314.54 ± 0.35	−7.92 ± 0.67
US	299.35 ± 0.21	323.13 ± 0.14	310.65 ± 0.17	−5.86 ± 0.13
MW	300.45 ± 0.15	324.12 ± 0.16	310.01 ± 0.16	−5.69 ± 0.13
UME	298.40 ± 0.22	319.49 ± 0.23	307.40 ± 0.46	−5.19 ± 0.32

Con: control, US: ultrasonic extraction, MW: microwave extraction, UME: ultrasonic-microwave synergistic extraction, T_on_: onset temperature (°C), T_end_: end temperature (°C), T_peak_: peak temperature (°C), ∆H: melting enthalpy (J/g).

## Data Availability

Data are contained within the article.

## Supplementary Materials

The following supporting information can be downloaded at: https://www.mdpi.com/article/10.3390/molecules28237746/s1, Figure S1: High-performance liquid chromatogram of five main taxanes. (A) standard substance, (B) sample after extract. 1, 2, 3, 4, 5 were 10-DAB III, Baccatine III, 10-DAT, Cephalomannine, and Paclitaxel, respectively.; Table S1: The regression equation and linear range of the five main taxanes.
