# Supplementary material for: Parameter Optimization of Ultrasonic–Microwave Synergistic Extraction of Taxanes from Taxus cuspidata Needles
Source: Molecules. 2023 Nov 24;28(23):7746. doi: 10.3390/molecules28237746 (PMC10708025; doi:10.3390/molecules28237746)
Supplement: Supplementary file 1 [file molecules-28-07746-s001.zip › molecules-2692905-supplementary.pdf]

Parameter Optimization of Ultrasonic-microwave Synergistic Extraction of Taxanes from *Taxus cuspidata* Needles

Zirui Zhao et al

Supplementary File

Supplementary Figure S1

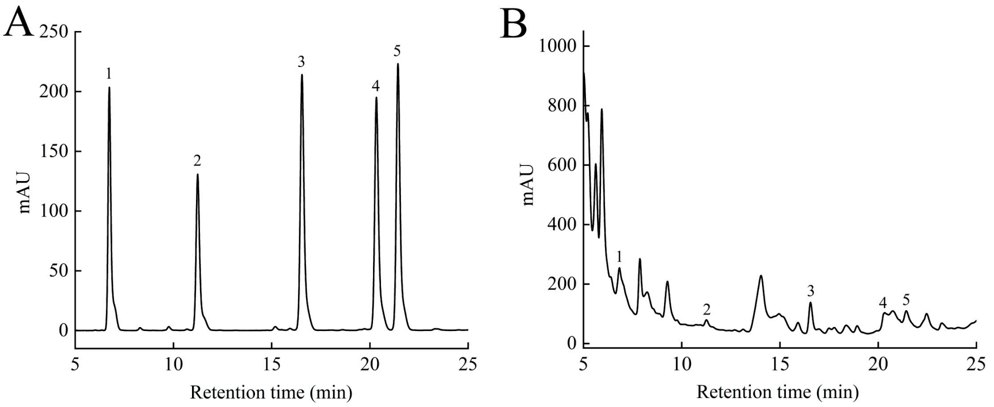

Figure S1. High-performance liquid chromatogram of five main taxanes. (A) standard substance, (B) sample after extract. 1, 2, 3, 4, 5 were 10-DAB III, Baccatine III, 10-DAT, Cephalomannine, and Paclitaxel, respectively.

Supplementary Table S1

Table S1. The regression equation and linear range of the five main taxanes.

Elution order	Compound	Regression equation	Correlation coefficient	Linearity range
1	10-DAB III	Y=-11853.87X+23196.98	0.9999	1-100
2	Baccatine III	Y=-17507.92X+18122.44	0.9996	1-100
3	10-DAT	Y=-15688.62X+31103.55	0.9999	1-100
4	Cephalomannine	Y=-2962.391X+26239.53	0.9934	1-100
5	Paclitaxel	Y=-14899.99X+32387.80	0.9999	1-100
